# Acute lumbar polyradiculoneuropathy as early sign of methotrexate intrathecal neurotoxicity: Case report and literature review

**DOI:** 10.1002/ccr3.2026

**Published:** 2019-02-19

**Authors:** Carmen Montejo, Judith Navarro‐Otano, Gerard Mayà‐Casalprim, Michela Campolo, Jordi Casanova‐Mollá

**Affiliations:** ^1^ Department of Neurology, EMG and Neuropathic Pain Unit Hospital Clínic Barcelona Spain; ^2^ Institut d´Investigacions Biomèdiques August Pi i Sunyer (IDIBAPS) University of Barcelona Barcelona Spain

**Keywords:** chemotherapy, F wave, methotrexate, neurotoxicity, polyradiculoneuropathy

## Abstract

Acute paraplegia after treatment with intrathecal methotrexate requires a complete spinal cord neuroimaging as well as electrodiagnostic examination. The absence of lumbosacral F waves motor responses without demyelinating findings may indicate early direct root toxicity. Early electromyography (EMG) screening could be a valuable tool for detecting peripheral neurotoxicity.

## 1 INTRODUCTION

Acute paraplegia after treatment with intrathecal methotrexate requires spinal cord neuroimaging and electrodiagnosis. We describe two cases of methotrexate neurotoxicity, with absent lumbosacral F wave as early direct motor root toxicity. Due to the poor prognosis observed in eleven cases reviewed, this finding provides a rationale for an early screening.

An early diagnosis of acute motor impairment during chemotherapy may be relevant to prevent paraplegia, especially in patients with hematologic neoplasia. Intrathecal (it) chemotherapeutic regimens such as methotrexate (MTX) combined with cytarabine arabinoside (Ara‐C) are used as treatment and prophylaxis of central nervous system (CNS) leukemia.^1^, ^2^ Neurological complications of this chemotherapy vary from asymptomatic chemical arachnoiditis to stroke‐mimics, leukoencephalopathy, myelopathy, and/or cauda equina syndrome.^3^, ^4^, ^5^, ^6^, ^7^ MXT is a dihydrofolate reductase inhibitor that induces experimental demyelination.^8^ Despite the mechanisms of MXT toxicity are unclear, some authors suggested to be dose dependent and related to a possible reduce clearance^9^ in cerebrospinal fluid while others related to a local depletion of folate due to MTX consumption folate^10^ and the improvement after folic acid supplementation.^11^, ^12^

Electrophysiological studies may help in useful in this setting. Among all the findings, the F wave latency measures the conduction time in motor fibers from the stimulus site to the spinal cord and subsequent return to the peripheral site of recording. Its absence provides evidence of conduction block of anterior rami at specific root level and has been considered specific for demyelination.^13^

We reported two cases of acute neurotoxicity related to MTX‐it with an early neurophysiological screening that help to define poor prognosis and review of previous clinical and neurophysiological cases published in the literature.

## 2 MATERIALS AND METHODS

Two patients were referred to the Neurology Department of Hospital Clinic in Barcelona. The neurophysiological tests were performed with Dantec KeyPoint Net G4 electromyograph (Natus Medical Inc., Pleasanton, CA, USA) following conventional methods for routine electrodiagnostic testing. The study was approved by the Ethical Committee of the Hospital Clinic of Barcelona, and all patients gave their written informed consent which included image permission for publication.

### 2.1 Case report 1

**Figure 1:**
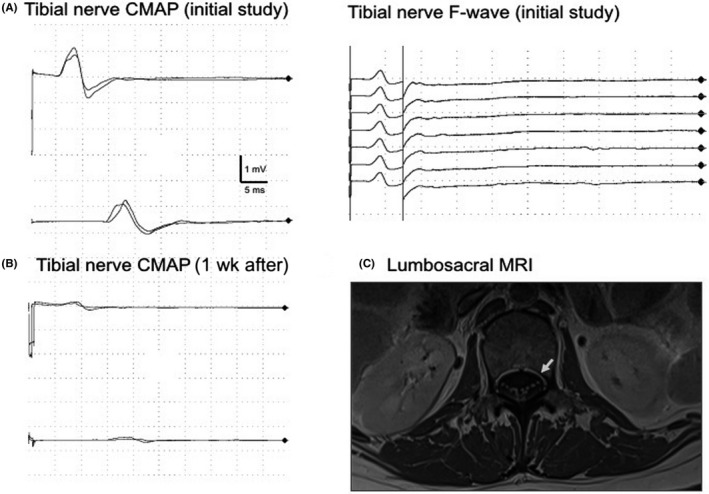
Case 1: Nerve conduction study at the initial phase (A) showed a relatively preserved compound motor action potential (CMAP) together with absent proximal F wave for the same nerve (proximal motor conduction block). The same study was repeated 1 wk after (B) and it showed severely decreased amplitudes without latencies delay at lower limbs. Case 2: Lumbosacral magnetic resonance imaging (MRI) at T1‐weighted with gadolinium in sagittal sequences showed an enhancement of spinal cord roots at L2 level (C, arrow)

A 58‐year‐old man with high‐grade B lymphoma received treatment with cyclophosphamide and rituximab, and triple intrathecal therapy (MTX, Ara‐C, and dexamethasone) as CNS prophylaxis. He received three doses of MTX‐it, with a total dose of 36 mg in three non‐consecutive days. Ten days after the last lumbar puncture, he complained with lower limb weakness, which evolved into paraplegia and urinary retention. Neurological examination revealed absence of deep tendon reflexes in lower limbs and a sensory level at T1. Cerebrospinal fluid (CSF) parameters were within normal limits. Nerve conduction studies (NCS) and electromyography (EMG) performed 1 week after neurological onset showed the absence of the F wave in both lower limbs with a minimal amplitude decrease and normal latency in CMAP responses suggesting a lumbosacral polyradiculoneuropathy. No abnormalities were found in upper limbs (see Table 1 and Figure 1A,B). Lumbosacral magnetic resonance imaging (MRI) with gadolinium revealed no abnormalities. MTX‐it treatment was stopped and the patient was empirically treated with intravenous methylprednisolone without improvement. One week later NCS and EMG studies showed a dramatic decrease of motor amplitudes with relatively normal latencies in peroneal and tibial posterior nerves of both sides (<1 mV) and moderate denervation in proximal and distal muscles of lower limbs (see Table 1). Thoracic spinal cord MRI revealed no abnormalities 2 months from onset. No improvement was observed after 6 months of physiotherapy and he remained with flaccid paraplegia and sensory level.

**Table 1:** Results on nerve conduction and EMG studies

	Patient 1		Patient 2	
	Onset	After 1 wk	Onset	After 3 wk
Median nerve				
Motor distal latency (≤3.9 ms)	3.2	3.1	2.9	2.8
CMAP amplitude (≥6.0 mV)	7.4	7.4	15	13
Motor CV (≥50.0 m/s)	60	61	60	61
SNAP amplitude (≥21 µV)	23	22	ND	26
F wave latency (≤31 ms)	29	29	24	23
Peroneal nerve				
Motor distal latency (≤5.0 ms)	4	0	0	0
CMAP amplitude (≥2.0 mV)	1.1	0	0	0
Motor CV (≥42.0 m/s)	45	‐	‐	‐
SNAP Amplitude (≥4.0 µV)	6	6	8	2
F wave latency (≤57.0 ms)	NONE	NONE	NONE	NONE
Tibial Posterior nerve				
Motor distal latency (≤6.0 ms)	5.5	5.2	5.1	0
CMAP amplitude (≥3.0 mV)	2	0.3	1	0
Motor CV (≥38.0 m/s)	40	41	52	‐
F wave latency (≤57.0 ms)	NONE	NONE	NONE	NONE
Sural nerve				
Sensory distal latency (≤3.0 ms)	2,6	ND	2,5	2.6
SNAP amplitude (≥7.0 µV)	8	ND	25	20
Sensory CV (≥38.0 m/s)	53	ND	62	52
Tibialis Anterior				
Fibrillation potentials	+	+++	++	+++
MUP recruitment	R	R	R	R
Quadriceps				
Fibrillation potentials	+	+++	++	+++
MUP recruitment	R	R	R	R

1 wk, one week; CMAP, compound muscle action potential; CV, conduction velocity; MUP, motor unit potential; ND, not done; NONE, no response; R, reduced; SNAP, sensory nerve action potential; Fibrillation qualitative measurement: + minimum; ++ mild; +++ moderate; ++++ severe.

### 2.2 Case report 2

A 26‐year‐old woman previously diagnosed with acute lymphocytic leukemia in 2017 was treated with dexamethasone, vincristine, MTX, and PEG‐asparaginase as consolidation therapy and MTX‐it plus dexamethasone for CNS prophylaxis. She received five doses of MTX‐it (total dose of 60 mg) over 5 months. Eight days after the last MTX‐it injection, she developed urinary retention followed by lower limbs weakness which progressed to severe flaccid paraplegia and areflexia. CSF study revealed high protein concentration (96.4 mg/dL), with normal cell count. NCS and EMG studies at onset (10 days) and follow‐up (22 and 90 days) suggested a motor lumbosacral polyradiculoneuropathy (see Table 1). Lumbosacral MRI demonstrated gadolinium enhancement of the anterior roots of the cauda equina at onset (see Figure 1C) and atrophy of dorsal columns and conus medullaris at follow‐up (90 days). MTX‐it was stopped but the patient showed no clinical improvement. After 6 months of physiotherapy, she persisted with flaccid paraplegia and EMG showed severe denervation of lower limb muscles.

## 3 DISCUSSION

To compare our findings with those of previously reported cases, we performed a comprehensive PubMed search using the terms “neurotoxicity and methotrexate” and identified 11 cases. Relevant information including clinical and neurophysiological findings is summarized in Table 2. We did not include a series of 11 cases treated with MXT‐it reported by Bleyer et al^9^ and a series of 11 cases reported by Geiser et al^3^ because of lack of clinical and neurophysiological information.

**Table 2:** Review of clinical and other characteristics of reported cases with acute motor neuropathy related to MTX‐it treatment

Case	Sex	Age (y)	Etiology	Drug intrathecal	Doses (mg)	Symptoms	CSF proteins	MRI	NCS	F wave	Recovery, treatment
Gagliano^14^	M	29	Leukemia	MTX	192	Motor (LL) Sensory level T11, Urinary retention	‐	‐	‐	‐	Good, DXT, Fol
Norman^15^	M	3	ALL	MTX	‐	Motor (UL + LL) Paresthesias	Normal	‐	Axonal motor neuropathy	‐	Improved, MPS
Koh et al^5^	M	5	ALL	MTX	36	Motor (LL), Urinary retention	Elevated	Enhancement anterior roots	Axonal motor neuropathy	‐	Good, MPS
Koh et al^5^	M	4	ALL	MTX	132	Motor (LL)	Normal	Enhancement anterior roots	Axonal motor neuropathy	‐	Good, MPS
Koh et al^5^	M	1	ALL	MTX	48	Motor (LL)	Elevated	Enhancement anterior roots	Peroneal axonal neuropathy	‐	Improved, MPS
Anderson^16^	F	3	ALL	MTX Ara‐C	‐	Motor (UL + LL)	Elevated	Enhancement anterior roots	Axonal multilevel polyradiculopathy	Absent	Good, IgIV
González‐Pérez^17^	M	19	ALL	MTX Ara‐C	24	Motor (LL), Bladder dysfunction	Elevated	Normal	Axonal motor neuropathy	‐	Death, IgIV
Pascual^18^	M	30	ALL	MTX	12	Motor (LL), Hypopallesthesia, Urinary retention	Elevated	Normal	Lumbosacral polyradiculopathy	Absent	No change, IgIV
Santos‐García^19^	M	45	ALL	MTX Ara‐C	36	Motor (LL)	Normal	Enhancement anterior roots	Lumbosacral polyradiculopathy	‐	Good, MPS
Lee^20^	F	25	ALL	MTX Ara‐C	12	Motor (LL), Sensory loss, Urinary retention	‐	Enhancement anterior roots	Motor neuropathy	‐	No change, MPS
Park et al^11^	M	58	Lymphoma	MTX Ara‐C	15	Motor (LL), Sensory level L2, Urinary retention	Normal	Normal	Lumbosacral polyradiculopathy	Absent	No change, ‐
Montejo (this article)	M	58	Lymphoma	MTX Ara‐C	36	Motor (LL), Sensory L1 level, Urinary retention	Normal	Normal	Lumbosacral polyradiculopathy	Absent	No change, ‐
Montejo (this article)	F	26	ALL	MTX	60	Motor (LL), Urinary retention	Elevated	Enhancement anterior roots	Lumbosacral polyradiculopathy	Absent	No change, ‐

ALL, acute lymphoblastic leukemia; Ara‐C, Citarabine; B‐NHL, B Non Hodgkin Lymphoma; CSF, cerebrospinal fluid; DXT, dexamethasone; F, female; Fo, folinic acid; IgIV, immunoglobulins intravenous; LL, lower limbs; M, male; MPS, methylprednisolone intravenous; MTX, Methotexate; NCS, nerve conduction studies; UL, upper limbs; ‐, unknown or no treatment.

Neurotoxicity related to MTX‐it may affect central and peripheral nervous system. Our cases highlighted that a more selective motor nerve damage was possible in contrast to immune‐mediated mechanism observed in monoclonal antibody‐agents such as ipilimumab or pembrolizumab,^21^, ^22^ which produce a generalized pattern of acute motor neuropathy. The neurotoxic mechanism due to intrathecal administration of MTX seems to interfere in folates metabolism at spinal cord (dorsal columns and motor neurons). It may produce myelopathy^7^ with or without the involvement of proximal motor roots at the lumbosacral level preferentially within days to weeks and generally has poor prognosis. Our cases emphasize that the absence of motor F wave response, which basically depends on the conduction of the proximal motor roots is an early neurophysiological sign of neurotoxicity damage and appears even earlier than the reduction of CMAP amplitude in distal motor nerves. This is noteworthy since this effect may not be dose dependent (see Table 2) but it takes some time to appear. In fact, Grzelec et al^23^ conducted a prospective study where he recorded F wave in two time points (before MTX‐it exposure and 24 hours after) and did not observe significant changes. However, it has been described as very early sign in Guillain‐Barré syndrome between 4 and 10 days after clinical onset.^24^

Neurophysiological studies provide relevant information to characterize the pattern of nerve damage. As an example, demyelinating peripheral neuropathy observed in Guillain‐Barré syndrome or paraneoplastic‐related lymphoproliferative disorders^25^, ^26^, ^27^ is frequently diffuse. Otherwise, mono or multifocal vasculitic pattern seen in neurolinfomatosis provokes a focal and direct nerve infiltration.^28^

In cases such as acute or subacute paraplegia related to lumbar polyradiculoneuropathy, finding the underlying mechanism (neurotoxic or immune‐mediated) is challenging as it is observed in 4^15^, ^16^, ^17^, ^18^ out of 11 cases reported in Table 2. First, high protein concentration in the CSF may be present in both scenarios, and it was seen in six cases treated with MTX‐it. In all cases, CSF‐flow cytometry and quantitative polymerase chain reaction (PCR) of virus have to be considered to rule out infective or infiltrative aetiologies. Second, MRI gadolinium enhancement of lumbar nerve roots may not be useful to differentiate both mechanisms since it has also been reported in patients treated with MTX‐it, including one of our patients.^5^, ^16^, ^19^, ^20^ In addition, spinal cord MRI T2 hyperintensities have been described in large series of patients treated with MTX‐it.^7^ Cachia et al^7^ reported seven patients with clinical features of paraplegia and sensory deficits (saddle anesthesia and/or sensory level) and 71% showed T2 hyperintensities at more than one spinal level. Third, NCS findings such as chronodispersion, prolonged latency or absent of F wave have been considered specific for demyelination^29^ as occur in polyneuropathies like Guillain‐Barré syndrome or chronic inflammatory demyelinating neuropathy (CIDP).^24^, ^29^, ^30^ Regardless of the etiology, the F wave latency provides evidence of conduction block at anterior rami of root level (in this case, L5/S1 at lower limbs) and can also reflect segmental motoneuron pool excitability.^30^ Prolonged F wave latency and increased amplitude with dispersion have been described in patients with upper motor neuron syndromes.^30^ In cases of traumatic spinal block, Leis et al^31^ reported low persistence or absent F wave at acute phase after injury and increased F wave persistence in chronic stages. Thus, rostral spinal cord injury might produce postsynaptic changes (hyperpolarization) at caudal spinal motoneurons pool in early stages (several weeks). At follow‐up, NCS‐EMG revealed a progressive decrease of CMAP amplitude with denervation compatible with severe axonal degeneration. No other signs of demyelination were observed despite the absence of F wave. Therefore, the NCS‐EMG may be indistinct of polyneuropathy in a chronic stage. Overall, these findings emphasize the need to perform periodic NCS‐EMG from onset of clinical picture to help in the differential diagnosis process.

No standard recommendation exists for treating chemotherapy‐related neurotoxicity as underlying mechanisms are unknown and there is a lack of evidence based on clinical trials. Evidence based on few clinical cases treated with methylprednisolone (MPS) with folic acid and B12 vitamin supplementation (four out of six patients) showed clinical improvement. Despite good prognosis has been reported in 6 out of 10 patients treated with MTX‐it, our experience is far from good. This discrepancy may be related to different demographic features since we included only adult patients in contrast to previously reported cases that included also children (40%).

In conclusion, electrodiagnosis should be considered a valuable tool for screening of peripheral neurotoxicity by including proximal and distal motor conduction studies as necessary to detect early signs (absence of F waves) of lumbosacral motor roots damage due to MTX‐it. In addition, multicentre drug vigilance programs in patients treated with neurotoxic drugs as MTX‐it should be encouraged to better characterize risk factors.

## CONFLICT OF INTEREST

None declared.

## AUTHOR CONTRIBUTION

C. Montejo and J. Casanova‐Mollá: should be considered joint first author.
